# A retrospective analysis of the perceived impact of the COVID-19 pandemic on systemic barriers to success for university student parents

**DOI:** 10.3389/feduc.2024.1256454

**Published:** 2024-04-07

**Authors:** Khadeejah T. Franklin, Alexa J. Saval, Anne M. Cafer, Dana N. Reinemann

**Affiliations:** 1 Department of Psychology, University of Mississippi, Oxford, MS, United States,; 2 General Engineering Program, University of Mississippi, Oxford, MS, United States,; 3 Center for Population Studies, University of Mississippi, Oxford, MS, United States,; 4 Department of Biomedical Engineering, University of Mississippi, Oxford, MS, United States,; 5 Department of Chemical Engineering, University of Mississippi, Oxford, MS, United States

**Keywords:** student parents, COVID-19 pandemic, mental health, coping resources, higher education

## Abstract

**Introduction:**

Student parents, both undergraduate and graduate, face the difficult task of balancing their studies and raising children, and they are a population often neglected or forgotten by higher education administration. The COVID-19 pandemic enhanced already present issues student parents face through the implementation of virtual schooling, increased daycare costs and closings, staying home with sick children, and a lack of local support system, among others. Further, many student parents are graduate students who are performing research that requires physical campus space and equipment to fulfill their educational requirements, and their research progress come to a halt when the country locked down.

**Methods:**

This study explored the struggles student parents faced prior to the COVID-19 pandemic, what issues the pandemic exacerbated, and what new problems have since arisen. Participants completed surveys assessing the consequences of being a student parent during the pandemic, coping resources available to them, the effect of being a student parent during the pandemic on their mental health, and demographic information.

**Results:**

Prevalent themes include substantial declines in mental health, feelings of inadequacy in regards to both their parenting and academic abilities compared to their non-student parent peers, and a striking lack of resources or acknowledgement from their institution.

**Discussion:**

The survey results are framed within the social-ecological model to better understand the systemic implications of student parent conditions. Finally, we formulate a set of recommendations to higher education administrations to inform them about the unique struggles student parents face and suggest strategies for mitigation.

## Introduction

The group of students within higher education that also navigate the difficult role of parent are a relatively understudied group. Prior to the COVID-19 pandemic, there was a growing literature on higher education student parents (Wainwright and Marandet, 2010; Moreau and Kerner, 2015; Moreau, 2016a,b). This literature articulates several sets of challenges faced by student parents, namely in the United States and Great Britain. Broadly, the literature describes a subset of the higher education student population that faces situations unlike that of their non-student parent counterparts where they perform the challenging task of balancing their studies and raising children (Brooks, 2012; Moreau and Kerner, 2015; Sallee, 2015). The addition of the COVID-19 pandemic introduced many new problems for student parents and exacerbated existing systemic challenges. Yet, a limited number of studies have emerged exploring the differential impact of COVID-19 on this marginalized group of students (Abbas et al., 2021; Lin et al., 2022; Holmes and Nikiforidou, 2023; Nikiforidou and Holmes, 2023; Sallee and Yates, 2023). These studies focus on student parent experiences at the height of the pandemic. This article hopes to explore, in tandem with these previous studies, the broad set of challenges experienced by student parents, but over the course of COVID-19 and the brief “post”-pandemic period as students were being reintroduced to campuses.

## Literature review

The population of students who are also parents might seem small to non-student parent counterparts and higher education administration due to their lack of visibility, but literature shows that “approximately 3.8 million (22% of all) undergraduate students in the US are raising children” (Lin et al., 2022; Todd, 2023). The literature demonstrates that prior to the COVID-19 pandemic, a majority of student parents struggled to cope with existing financial insecurity due to the cost of raising children in the current economic climate (Gerrard and Roberts, 2006; Gault et al., 2018). One source reports that over two thirds of the student parent population live in or near poverty (Cruse et al., 2020), demonstrating the financial strain experienced by student parents. Further, the literature also confirms that the majority of student parents experience financial strain due to childcare costs (Demeules and Hamer, 2013; Long, 2017; Sallee and Cox, 2019; Ajayi et al., 2022; Sallee and Yates, 2023). Graduate student parents at Stanford faced an existing affordability crisis even before the pandemic (The Stanford Daily, 2020), and the addition of the pandemic served to worsen the existing insecurity.

In addition to financial insecurity plaguing student parents, the literature also confirms a lack of resources in place to aid student parents with the unique issues they face. Historically speaking, higher education institutions are designed to serve students whose primary role is being a student and who likely does not have competing priorities of that magnitude (Medved and Heisler, 2002; Roy et al., 2018; Manze et al., 2021; Reed et al., 2021). The literature finds that compared to traditional college students, student parents are navigating systems that fail to account for the diversity of their needs (Estes, 2011; Brooks, 2013; Manze et al., 2021; Ajayi et al., 2022; Briegel et al., 2023; Sallee and Yates, 2023). Further, in a study conducted of student mothers, it was found that “89% of students could not identify the support available to student parents” and the department chairs themselves could not identify any university services in place to help student parents cope (Holm et al., 2015). The lack of resources available to student parents both before and during the pandemic places student parents at a disadvantage from their non-student parent counterparts, putting them at risk of falling behind academically. In cases where resources are available, the translation of their availability to student parents is not well-defined or communicated. Lin finds that “approximately 75% of student parents are uninformed that their financial aid could be increased to account for childcare costs” (Lin et al., 2022). The existence of resources for student parents alone is not enough to mitigate the challenges they face; they must also be clearly and widely communicated with student parents.

Mental health is also a particular challenge for student parents. While attending college or university can be difficult for any student, and mental health is of particular concern, the additional stress of parenting creates unique and acute mental health challenges for student parents (Brooks, 2015; Abbas et al., 2021; Cho et al., 2021). This stress has many sources, but unique to student parents is the guilt of prioritizing one’s needs above social expectations of parenting, particularly for mothers (Brooks, 2015; Moreau, 2016a,b).

The pandemic greatly disrupted the lives of student parents and, in addition to exacerbating those already articulated in the literature, introduced new hurdles for student parents. The transition to remote activities during the pandemic forced student parents to juggle working from home with taking care of their children (Moreau and Kerner, 2015; Lin et al., 2022; Nikiforidou and Holmes, 2023; Todd, 2023). Due to the loss of uninterrupted, designated work time while working from home, student parents often felt guilty for not spending enough time with their children or on their work (Nikiforidou and Holmes, 2023; Todd, 2023). Student parents also struggled finding childcare during this time as daycare closures were frequent occurrences, and this greatly impacted the academic performance of many student parents (Sallee and Yates, 2023). Further, the burden typically falls more on student mothers rather than student fathers (Sallee and Yates, 2023). The literature finds that “instead of writing papers, [student mothers] are likely to devote time to homeschooling children and doing household chores” (Staniscuaski et al., 2020).

The goal of this research is to explore the unique barriers student parents faced in the COVID-19 pandemic context and provide some nuance to existing recommendations for higher education systems for supporting these student parents in a post-pandemic state. The literature demonstrates a clear call to action in order to help student parents navigate the pre-existing challenges they experience and provide targeted support to help them cope with the unforeseen challenges introduced by the pandemic. The motivation behind these measures is to not only improve the academic success of student parents, but also the overall well-being of student parents by prioritizing their needs.

### Social-ecological model

A conceptual framework that will aid in understanding the context of student parent experiences during the pandemic is the social-ecological model introduced in 1979 by Bronfenbrenner (1979). The social-ecological model is broken into four overlapping categories—individual, relationship, community, and societal factors—in order to understand how the social environments of individuals affect each other in a dynamic fashion and how those emergent interconnections shape one’s development (Bronfenbrenner, 1979; ATSDR, 2015; Kilanowski, 2017). On the individual level, personal history and biological factors are viewed in order to help understand why a person feels or acts a certain way (Kilanowski, 2017). The identity of the participant is used in order to help fill this space with important information such as their age, income, educational background, gender, ethnic affiliation, social class, and religious affiliation. For the purposes of this study, the individual is the student parent. The relationship category focuses on how important close relationships are to an individual and how they can affect a person’s response to a situation (Kilanowski, 2017). For student parents, this includes their familial relationships, such as to their child, spouse/partner, and other family members; their peer relationships, such as to friends or other student parents; and their academic relationships, such as to their professors, academic mentors, and research advisors (Briegel et al., 2023). The community level includes the broader areas in which an individual has these social relationships, such as schools, neighborhoods, workplaces, and religious places (Kilanowski, 2017). A student parent’s community may look vastly different from another student parent depending on the resources and relationships they have. In theory, there are groups of student parents within various institutions that would constitute a “student parent community”; however, the lack of organization and knowledge of this student population by higher education administrations is limited at best, rendering this community invisible in many instances (Todd, 2023). The last category of this model includes broad societal factors that influence an individual, such as healthcare, finances, political policies, and education that can affect how a person makes decisions regarding their community, relationships, and individual goals (Kilanowski, 2017). For a student parent, this could include perceptions of student parents by society, traditional gender roles, laws and policies in higher education, and government regulations regarding the COVID-19 pandemic (Briegel et al., 2023; Sallee and Yates, 2023).

The social-ecological model (SEM) correlates well with this research because each branch is incorporated into the participants’ lifestyles and have an interconnected influence on the outcome of the other (Ajayi et al., 2022). Each student parent has individual identities that caused them to be affected by the pandemic mentally and have different experiences from their peers. They also have relationships with their significant others, professors, advisors, daycare teachers, and family members that can influence mental, physical, and social shifts that propagate to multiple areas of their lives (Ajayi et al., 2022). Student parents are a community itself, but each student also belongs in a community uniquely defined by their social and familial relationships, background, culture, and personal experiences (Sallee and Yates, 2023). In addition, societal factors in the student parents’ lives shift their ability to adapt to the changes that they had to face when the COVID-19 pandemic hit, such as parents contracting COVID-19, needing to take care of a sick loved one, or the resources available to them (Todd, 2023). Educationally, these student parents had to change their methods and environment for learning (Todd, 2023). Further, multiple studies surrounding student populations utilize the SEM to contextualize the results of their studies. Lisnyj et al. used the SEM to explore which factors affect post-secondary students’ stress and academic success (Lisnyj et al., 2021). The SEM has also been used to explore how different levels of the model affect COVID-19 infection preventative behaviors (Vilme et al., 2022). Another study that has ties between the SEM and college students breaks down the barriers and enablers to a healthy diet in college students into the individual level, social level, and community levels in order to explain how these factors affect a college student’s experience (Sogari et al., 2018). In this study, the SEM will serve as a platform for understanding the complex emergence of barriers that arose for student parents during the pandemic and for formulating recommendations to higher education officials to aid student parents under “normal” conditions but also those in times of crisis.

## Materials and methods

This mixed-methods exploratory study utilized a community-based participatory approach to investigating student parents’ views and experiences during the COVID-19 pandemic and their return to campus. The research team first engaged with key stakeholders on the university campus, namely student parent campus leaders in a focus group format. During this engagement, student parent leaders expressed an interest in collecting data that would help them and university administration understand key concerns or challenges student parents navigated on campus and how those challenges might be exacerbated by the COVID-19 pandemic. During these focus groups, student parent leaders and the research team developed the research questions that ultimately shaped the project. This engagement was the basis for the preliminary survey developed by the research team. The survey was vetted by student parents before deployment.

This specific combination of mixed methods—focus group followed by survey—has been employed successfully by other studies of student parents before and during the pandemic. This approach allows a research team to engage a relatively small population in a robust way and to be inclusive of their limited ability to commit time to participating in a study (King, 2022; Briegel et al., 2023; Holmes and Nikiforidou, 2023; Nikiforidou and Holmes, 2023; Todd, 2023). Todd purports that it can be “challenging to engage with an ‘invisible’ cohort,” and there are “no provisions at the institutional level to gather data on student parents” (Todd, 2023). In addition, online surveys enable data collection due to irregularities in childcare availability due to pandemic restrictions and/or illness and grant flexibility in finishing the survey due to school and parent responsibilities (Holmes and Nikiforidou, 2023; Nikiforidou and Holmes, 2023). Thus, our study approach was used to yield a complementary view of student parents’ various experiences while providing an inclusive and accessible format to accommodate their unique needs (Johnson and Onwuegbuzie, 2004; Shorten and Smith, 2017; Nikiforidou and Holmes, 2023).

### Survey design and administration

The survey, co-developed by the research team and student parents, was anonymized and distributed online via Qualtrics. The online approach was deemed most prudent during the COVID-19 pandemic as it also provided survey participants a flexible timeline for completion given the many demands of their multiple roles (Holmes and Nikiforidou, 2023; Nikiforidou and Holmes, 2023; Todd, 2023). Anonymity was important to mitigate feelings of pressure or judgment by having someone watch them complete a task or answer questions (Su, 2022). Also based on previous literature focused on student parents, as well as stakeholder engagement, anonymity was crucial given the real or perceived potential consequences of speaking out about unfair or unjust practices within the student parents’ working and learning environments (Su, 2022).

The survey was comprised of 35 questions broken down into five categories: (1) Demographics, (2) Effect on Mental Health, (3) Pandemic Consequences, (4) Coping Resources, and (5) Recommendations. These categories were based on engagement with stakeholders and consultation of the existing literature on student parent experiences. The project received IRB-approval from University of Mississippi’s Institutional Review Board (protocol 22x-096).

Demographic questions included: participants’ age, relationship status, self-reported gender, ethnicity, employment status, family’s COVID-19 vaccination status, and current educational level (undergraduate or graduate level). The number of children and the individual’s family financial status were also gathered during this portion of the survey.

The Effects on Mental Health section gave the participants an opportunity to describe how the pandemic affected their mental health. The Pandemic Consequences section focused on changes student parents experienced in childcare decision-making and how they handled their school obligations due to the pandemic. Uniquely, and importantly, this section sought to collect data to compare experiences at different snapshots in time across a novel global pandemic, including during the height of the pandemic and upon return to in-person operations. The next set of questions focused on coping resources student parents received or did not receive while being a student parent. The final section of the survey was a qualitative open-ended question for student parents to identify potential solutions or provide additional context that might further clarify their responses.

It took participants between 10 and 15 min to complete the survey, and there was no financial compensation involved for completing the survey. Given the dearth of literature on the impact of COVID-19 on student parents, this survey is exploratory. In order to reach a broad audience in an online only environment, the survey was administered via the University of Mississippi’s email system. Additionally, the survey was sent to several other [Southeastern Conference: athletic conference] schools for consideration by the student affairs personnel for dissemination to their student body, as well as through the Twitter social media platform. Inclusion criteria for the survey included: (a) being 18 years of age or older, (b) being a student parent, and (c) being willing to completely finish the survey. If a respondent selected that they were under the age of 18 and/or not a student parent, then the survey automatically ended for them. For the purposes of this study, “student parent” was defined as anyone who currently had a minor dependent living with them.

### Analysis of survey data

The small sample size and the exploratory, rather than explanatory, nature of the study supported the use of descriptive statistics (Maguire and Delahunt, 2017). Qualitative responses were analyzed using iterative qualitative thematic coding within the context of the social-ecological model. The qualitative thematic coding approach identified patterns across the qualitative survey responses to provide a systematic interpretation of participants’ experiences and perceptions (Tarzia et al., 2023). The open-ended responses were analyzed using Braun and Clarke’s six stages of thematic analysis: familiarization with the data, generating initial codes, searching for themes, reviewing themes, defining and naming themes, and writing the report (Braun and Clarke, 2006). Illustrative quotes from the qualitative portion of the survey that speak directly to survey responses were incorporated into the results section for that set of questions.

SEM’s use to guide inquiry is documented in several cases related to students and higher education (Jack et al., 2019; Dotson et al., 2022; Wright, 2022). Jack et al. used SEM to guide inquiry, thematic coding, and analysis to provide insight into the perceptions of refugee student well-being within higher education, as well as for the professionals working with them (Jack et al., 2019). Wright investigated sexual assertiveness of undergraduate women in a southeastern US higher education institution, and Dotson et al. performed a qualitative study on US college youth online learning during the pandemic, both using an SEM guided, qualitative thematic coding approach (Dotson et al., 2022; Wright, 2022). Based on this previous use of SEM-guided analysis and qualitative thematic coding, we employed this complimentary framework and analytical approach to understand student parents’ experiences and perceptions during the pandemic.

### Sample population

The survey received 35 responses, ranging from 18 to 40+ years of age. Because the survey was anonymous, the research team was unable to determine the kinds of institutions represented in the survey. Responses were collected over 3 months from November 2021 to January 2022. The majority of respondents were female (*N* = 34) and white (*N* = 29). Educational backgrounds varied. Of the responses received, ten participants had a Bachelor’s degree, 22 had a Master’s degree, and one had recently received their Doctoral degree. All but one of the participants was currently enrolled in their university as a graduate student. Twenty-three of the 35 participants were not employed outside the university, and 23 were receiving an assistantship of some kind.

Most participants had one child (*N* = 21), while 14 participants had 2 or more children. Child ages ranged from infants (<1 year) to preteens (11 years). Thirty-three respondents were married or in a domestic or long-term partnership. Household incomes ranged from 10,000 USD to 350,000 USD. Only 3 respondents stated that their school-age children qualify for free or reduced lunch, and 2 of the respondents indicated that their family qualifies for SNAP (supplemental nutrition assistance program).

When asked about their COVID-19 vaccination status, 34 of the respondents stated that they have received the vaccine, and one selected that they preferred not to answer. We received the same responses to the question of whether or not their partner was vaccinated. Most respondents stated when their child is old enough, they would be willing to vaccinate their child as well with timing varying between when the vaccine was approved for emergency authorization versus full FDA approval.

### Study limitations

There were only 35 respondents, but given the challenge with engaging marginalized groups in higher education, the research team expected low participation (Todd, 2023). Additionally, the sample was not socioeconomically diverse, as most respondents were married and had a substantial income. However, many points of stress and questions about parenting traversed socioeconomic lines. The exploratory nature of the study, the sample characteristics, and low response rate make this study ungeneralizable, but still offers important insights into student parents during the COVID-19 pandemic and could serve as a starting point for future research.

## Results and discussion

### Pandemic consequences

Student parents faced many challenges prior to the pandemic, and the emergence of COVID-19 served to exacerbate these challenges and present new obstacles. With daycare closures and quarantines a regular occurrence, student parents were faced with the difficult task of balancing academic roles with parental roles during their usual hours of work. From the survey results, student parents reported that while working from home, they were less productive because they did not have designated, uninterrupted work time and also felt that they had to work outside of regular office hours (Monday–Friday, 9 a.m. to 5 p.m.) due to balancing childcare in order to meet their goals. Holmes and Nikiforidou found that 30% of their student parent respondents had to work during “anti-social” hours (Holmes and Nikiforidou, 2023). Moreover, student parents also reported that they believe their quality of work (course work, research, etc.) has suffered due to lack of childcare at home. Many student parents suggested that the most difficult part of working from home during the pandemic was balancing childcare with their academic work. One participant writes,

“Working while teaching children from home was not just unproductive, it was impossible. I have neurotypical children and a neurodivergent, special needs child. Helping them with schoolwork put me in the position of being a special education teacher, a regular teacher, IT support, therapist, mom, and PhD candidate. I am an enormously motivated, efficient person generally, but I do not know anyone who could have effectively balanced all of this.”

Other student parents agreed with this sentiment and felt that they could not devote themselves to one role, but instead had to take on multiple roles while working remotely with children at home. Because of this, student parents faced consequences in their relationships with those around them during the pandemic. The survey results revealed that many student parents felt a shift in their parenting styles and a change in their relationship with their children during this time. One participant writes,

“I am more reactive and less patient and working while my daughter was at home felt negligent”

when describing how working from home affected a relationship.

Many participants also reported that the COVID-19 pandemic would delay their thesis/dissertation completion and expected graduation. Further, a Likert scale question revealed that the majority of student parent respondents “somewhat agree” or “strongly agree” that working in academia during the pandemic made them consider a career change. This, unfortunately, aligns with and accents trends of females leaving academia upon reaching the stage of parenthood (Mirick and Wladkowski, 2018; Fulweiler et al., 2021). Together, these multiple exacerbations by the pandemic clearly impacted student parents not only in the moment, but also had the potential to change the trajectory of their career.

While the scope of this research does not seek to analyze the gender roles among student parents with spouses/partners, gender roles did also affect student parents during this time. Holmes and Nikiforidou reported that nearly 80% of their student parent respondents had perceptions that mothers and fathers experienced the pandemic lockdowns differently, with their most frequent response being that mothers experienced more pressures during lockdown than fathers (Holmes and Nikiforidou, 2023). In our study, a student mother found most difficulty with:

“having to take on the brunt of online schooling and child care because of gender norms in our household but also mostly because my spouse is the one with a job that pays the bills, my stipend does not, so his work took precedence over mine, every time. I had to drop a class then make it up over the summer. I was not able to submit to any conferences or complete research outside of class. I wasn’t able to read or give fully to any of my courses. I have been set back at least a year of graduation, which means finding funding for an extra year. It was difficult to focus on my schoolwork while my kids were struggling with school and mental health issues from being home.”

One benefit reported by student parents of working from home was that less time was spent commuting to and from work/school, which allowed student parents more time and flexibility. However, these benefits do not outweigh the negative consequences student parents faced due to the COVID-19 pandemic.

### Individual

In the context of the SEM model, survey responses indicate that while the manifestation of COVID-19 challenges were seen at the individual level (reduced productivity, reduced work quality), the root cause of these were related to society norms, relationship dynamics, and constraints within their larger academic and student communities (Wiese and Stertz, 2022; Holmes and Nikiforidou, 2023; Todd, 2023).

### Relationship

The survey showed that consequences of being a student parent also included dealing with partner relationship dynamics. When asked if they felt their partner contributed to balancing child care obligations during the pandemic, most of the respondents selected that they somewhat or strongly agree with the statement. However, there were still some that disagreed. This distribution of responses is also reflected in what was reported in Holmes and Nikiforidou (2023). There were similar results when asked if their partner/spouse/significant other was able to help with childcare while working from home. The vast majority of the respondents, 97 percent, identified themselves as females, so these results are mostly coming from a mother’s view. This is interesting because mothers are usually viewed as the primary caretakers for children and domestic duties, even as mothers enter the workforce and the prevalence of two-income households has increased drastically (Fetterolf and Rudman, 2014). We asked an open-ended question that stated, “What concerns, if any, do you have about your parenting style and/or abilities during the pandemic?” A few of the respondents mentioned that they were forced to allow their children to have more screen time due to the fact that they were less patient with the child or needed to focus. One parent stated,

“I am much less effective as a parent during the pandemic because I am exhausted and juggling too many tasks all the time without clear breaks between them. It’s insane.”

This is important as the consequences that they are facing with their parenting styles are heavily influenced by the adaptations they have had to make to deal with the pandemic.

### Community

Survey responses discovered that the parents deal with lack of support of resources. The question regarding whether or not these parents participate in a student parent community in their department/at their institution for resources and support received more strongly disagree responses than any other category. Having support and resources is something that could have been helpful to many of these parents during their transition to working at home during the pandemic; however, many of them either did not know they existed or their schools did not have a community in place for them. Todd coined the student parent population as invisible due to the lack of organized student parent communities, as well as lack of prioritization or even acknowledgement by higher education administrations (Todd, 2023). Over half of the respondents strongly disagreed that their educational institution has provided adequate support or resources to student parents throughout the duration of the COVID-19 pandemic. Another consequence that these student parents had to endure is working outside of regular office hours. They were forced to work outside of the traditional 9 am – 5 pm because their children were not at childcare or school at this time. Due to schools and daycares being closed for months as the pandemic began or due to frequent quarantines when they were back in session, the parents were required to make sure the child or children received the care and education they needed to stay on track with their school curriculums. In addition, this led to constant distractions throughout the day that led to parents having to work late nights and early mornings. Nikiforidou and Holmes use Third Space Theory to explore being a parent during lockdown, being a student during lockdown, and the space between, leading to an emergence of such strategies described above to try and balance the demands of being a student and parent simultaneously during the pandemic (Nikiforidou and Holmes, 2023).

### Societal

One of the main societal factors contributing here is gender and the traditional roles that accompany them. As mentioned, a majority of the respondents were women. Society often emphasizes women taking on the familial roles of childcare, cooking, cleaning, and providing support for their significant other. The pandemic overwhelmed these roles even more because both parents were required to stay home in many cases resulting in the women having to do more around the house, which has also been observed in other studies of student mothers (Holmes and Nikiforidou, 2023; Nikiforidou and Holmes, 2023; Sallee and Yates, 2023; Todd, 2023). These added stresses may cause even more women to leave the workforce, reducing diversity in the workplace and within societal roles.

### Effects on mental health

The obstacles traditionally faced by student parents became more challenging during the pandemic. Many of these student parents have the responsibility of providing for their children, completing schoolwork, caring for themselves, and making sure to complete the tasks required for their jobs. Along with this accompanies significant impacts on mental health. The survey results highlighted the mental toll the pandemic had on student parents while working from home. From the “check all that apply” questions in this section, 78% of participants felt stressed about their course work performance, 89% felt stressed about their research progress, and 86% felt stressed about the pandemic’s damage in their community. Additionally, the majority of the survey respondents agreed that they felt guilty for not spending adequate time on school work/research and their children while being a student parent during the pandemic. One positive effect of working from home was the lessened financial strain as only 33% of respondents reported feeling stressed about money while working from home; however, the prevalence of married respondents and dual-income households should be considered here. Holmes and Nikiforidou also found that some student parents found the pandemic as a learning opportunity for how to juggle school and parenting, as well as enjoyed the additional time with family (Nikiforidou and Holmes, 2023). Regardless, 75% of student parents reported that they felt an overall decline in their mental health while working from home during the pandemic. When asked to describe how working from home affected their mental health, one participant writes,

“I’m constantly stressed about being behind on research and constantly overwhelmed by both research and home related tasks. I’m never caught up and never get a real break from either role.”

Similarly, another student parent writes,

“Focus, achievement are both down and that makes me feel guilty and worried about my future. I’m constantly considering dropping out to alleviate the stress.”

Other student parents responded in a similar manner expressing how they are battling feelings of *“isolation and depression and lack of control”* which can play a major role in their performance as both a student and a parent. Others have found additional negative descriptors of student parent experiences during the pandemic, including “too much,” “overwhelmed,” “suffocating,” “exhausting,” and “depleting” (Holmes and Nikiforidou, 2023).

In addition to the stress caused by working from home, student parents also had reservations about returning to in person work/school activities. From the survey results, only 21% of respondents reported that they feel less stressed because they were returning to in person work/school activities, and only a third of respondents felt that they would get more accomplished when returning to in person school/work. In addition to the productivity worries of returning in person, many student parents reported feeling stressed about child illness and school/daycare closures during this time. For example, while returning to in person activities, 94% of student parents felt worried that they would have to miss work due to self or child illness, and 79% of participants felt worried that their child might get sent home from school for spiking a fever regardless of COVID-19 positive status. These worries took a great toll on the mental health of student parents, and 49% of student parents felt that they could not fully focus on their work due to the acute stress of self or child illness occurring. Because of the prevalence of COVID-19 cases at the time, many student parents felt that vaccination could reduce the possibility of self or child illness occurring. The survey results revealed that 47% of student parents “strongly agreed” that vaccination of self would alleviate stress associated with returning in person, and further 53% of respondents “strongly agreed” that vaccination of their children would alleviate the stress of returning in person. When asked to describe how to work/school/in-person activities affected their mental health, one participant writes,

“The biggest thing that both helps but makes me stressed/worried is sending my unvaccinated kid to daycare. My kid has asthma. We spent time in PICU when he was a baby, rsv multiple times, etc. So a lot of this takes me back to those triggering events. So while sending him helps me work better it also is always on my mind and I worry. It’s just a constant struggle.”

Another participant described a similar worry as they write,

“Anxiety over my young unvaccinated children getting ill, but also this chronic knot in my gut, waiting for a classroom exposure and quarantine.”

Further, another student parent found some comfort returning to routine in-person activities as they write,

“It helped at first, but it also brought out the realities of having completely socially isolated myself for months on end. I felt a lot of anxiety being around others with new social covid rules, including rules imposed by the University (which at my institution were awful. We weren’t allowed to eat or drink inside any University building for over three months). So a lot of anxiety, but also feeling good to be returning to my usual job with (slightly) the same routine.”

The heightened fear and stress that student parents experienced due to the pandemic had a significant impact on their mental health. Dealing with constant worries can cause an individual’s performance and enthusiasm for parenting and/or academics to be dampened. These participants were asked if they felt as though they were at a disadvantage from other students because they are raising child(ren), and most respondents stated yes. A few of the respondents stated,

“Yes. I feel like other students can be more productive at home than parents, and yet the expectations are the same and no special accommodations are given.”

“Yes-those without children have so much more flexibility to take on activities such as teaching. Whereas I have to balance work with childcare.”

The majority of respondents similarly felt that they were at a disadvantage compared to their non-student parent counterparts and felt overwhelmed by the stressors of the pandemic without having adequate resources to cope with the precarious situation. Todd’s study also reflects these statements and emphasizes that a sense of belonging increases student parent success at a university (Todd, 2023).

### Individual

Mental health is often overlooked by individuals when they have other factors requiring their immediate attention. Many students experienced anxiety and stress due to the unpredictability of the pandemic. They did not know when or if anyone in the family might contract the virus or if they might lose their job or keep up with their schoolwork. The stress that this created negatively impacted their academic performance as well as their overall well-being. When the respondents were asked to describe how working from home affected their mental health some of the comments included,

“So much anxiety. Isolation. Loss of Motivation. Alcohol Abuse”

“Constantly stressed about being overwhelmed by both research and home related activities”,

and

“Made me less tolerable of people and anxious.”

Many other statements aligned closely in tone and theme with the ones listed. Overall, working from home during COVID-19 caused a great deal of stress for these student parents. They were stressed about taking care of their child and schoolwork, not getting enough sleep, and the possibility of a loved one getting COVID-19, to name a few. When asked to describe how returning to school/work/in-person activities affected their mental health, there were a mixture of comments. Some were more at peace because they did not have family there to distract them from completing work-related tasks. Yet others were even more stressed because they had to worry about sending unvaccinated children to school. The attitude toward mental health is usually that it can be dealt with when time permits. However, most people forget to set aside time to deal with their emotional well-being, and it became even more difficult with the pandemic for these student parents who already have little time for themselves at baseline.

### Relationship

These parents had to balance their children’s needs as well as their own. The needs for their children included the transition to remote learning. Many parents were forced to be their child’s teacher in order to make sure their child did not fall behind. Strains between the parent and child arose because these parents did not get the normal time away from their children for school and/or work in addition to the added stress of having to balance their own work with teaching their children. During the pandemic, everyone was required to figure out how to keep children entertained enough for them to try and get their work done. This is a problem for student parents’ mental health because they do not have the time to themselves that they need. This is also reflected in Nikifouridou and Holmes’ Third Space Theory analysis of student parents bridging the gap between student and parent identities and the lack of resources available to help someone construct and sustain that bridge (Nikiforidou and Holmes, 2023). Another relationship that affected these student parents’ mental health was their connection with their research or graduate advisor. Many of the respondents did not feel supported by their advisor. The necessary shifts between online, hybrid, and in-person would have been easier if they had more advisors and/or professors willing to provide help, support, and flexibility (Todd, 2023). If the student parent felt like their advisors did not care about them enough to move due dates and be understanding, then they mentally would have more anxiety and stress than before.

### Community

A major theme at the community level was the institutional vs. local community’s effect on the individual. Institutionally, these student parents were forced to continue to produce the same workload as required before the pandemic with the now added responsibilities of childcare and their child’s virtual schooling. Nikiforidou and Holmes state that almost 30% of the participants in their study made specific references to “being a parent during the day and being a student during the night or when children were asleep” (Nikiforidou and Holmes, 2023). In relation to their local community, school and daycare center closures created a difficult situation for student parents to balance with their own responsibilities. Multiple times during the pandemic, childcare providers and schools had to shut their doors when an outbreak of the virus occurred (Yavorsky et al., 2022). Children had to wait until a certain age to be able to get vaccinated; even once they reached it, many parents did not want their child to receive the vaccine due to the seemingly rushed nature of approval, pre-existing health conditions, or social pressures, among other reasons (Olusanya et al., 2021). For single student parents with no help, this caused an even greater threat to their schoolwork and research/graduate work because they would have to take time off in order to care for their child or children. Missed assignments and lack of focus caused many student parents’ grades to drop causing their mental health to decline. This decline came from the fact that they had to lose sleep and personal time in order to make up for this over-extended workload (Nikiforidou and Holmes, 2023; Todd, 2023). Sallee and Yates argue that student mothers in particular turned to internal, women-comprised networks to help navigate parenting and academics during the pandemic; however, that support network has to exist and be accessible for student parents, which may not always be the case (Sallee and Yates, 2023).

### Societal

Society has created a stigma around student parents that has been a topic of discussion for many years (Ascend at the Aspen Institute, 2021). One pressing topic is whether or not students with children can do as much as those without due to the extra responsibilities that come with being a parent (Briegel et al., 2023). Due to these biases and microaggressions, many student parents feel inferior to their non-parent student peers (Moreau and Kerner, 2015; Holmes and Nikiforidou, 2023). In addition, financial insecurity among student parents with the addition of inflation, supply chain shortages, reduced hours, and losing jobs strikes more complex issues that are difficult to manage while also being a student and parent (Moreau and Kerner, 2015; Tsurugano et al., 2021).

### Coping resources

The last section of the survey aimed to identify the coping resources available to student parents throughout the duration of the COVID-19 pandemic. Many student parents alluded to a lack of resources available to them during this time that made working from home even harder. From the Likert scale questions, 69% of student parents strongly disagreed that their educational institution or university provided adequate resources to them during the pandemic. When asked what type of resources their institution provides for student parents, one participant writes,

“None. No parental leave. No committees. Absolutely nothing.”

Another participant conveyed a similar sentiment as they write,

“This is a thing? My advisor being a woman with children herself was the saving grace for my experience the last two years.”

In addition to the clear lack of resources available to student parents during the pandemic, 83% of student parent respondents strongly or somewhat disagreed that established student parent communities existed at their institution that they could participate in for resources and support. Some participants reported that they did not know of any other student parents in their department that they could contact for support. For many student parents, even knowing of someone else in a similar situation can provide comfort during difficult times. One student parent writes,

“The other parents in my cohort have been my lifeboat the past couple of years…”

demonstrating how knowing other student parents can help student parents feel less isolated during this time (Sallee and Yates, 2023; Todd, 2023).

Despite the lack of institutional resources provided for student parents during the pandemic, participants reported periods of grace while working from home. Some student parents found that while working from home, instructors and research advisors were understanding of delays and unmet deadlines due to childcare obligations or disruptions. Additionally, some participants agreed that research advisors and instructors communicated effectively with them on a regular basis. Upon returning to in-person, student parents found that research advisors and instructors were still understanding of delays and unmet deadlines due to childcare disruptions. Also, while working from home, 76% of participants had a significant other that was capable of helping with childcare, and but only a fraction of the student parents felt that their partner contributed to balancing childcare obligations during the pandemic, with the reminder that all but one respondent identified as a female. Gendered differences in parenting responsibilities among student parents have been previously reported as well (Holmes and Nikiforidou, 2023; Sallee and Yates, 2023).

The use of technology also played a key role in whether or not student parents were able to work effectively from home during the pandemic. From the survey, 71% of student parents found that the use of online technology to provide online learning and remote meetings helped them succeed during periods of remote work.

When asked what changes should be made in order to benefit parents that are also university students, most student parents strongly suggest some form of affordable on-campus childcare to aid them with childcare disruptions and obligations. Student parents also wish for more flexibility with deadlines and a greater understanding of the precarious situation student parents face. One student parent suggests,

“Flexible deadlines and an understanding to have the camera and mic off during meetings so I can do things like breastfeed/bounce a baby.”

Another student parent writes, asking for,

“Better clarity and knowledge when it comes to what ways the university can be flexible with us and make those policies universal vs instructor by instructor, and a better understanding of what parents are actually dealing with”

Student parents wish not only for resources to be more available to them, but also to be more effectively advertised in order for them to utilize the resources being offered. Many student parents were not aware of the existence of student parent support groups and better clarity can help them benefit from these resources. Holmes and Nikiforidou analyzed multiple university student support pages and found that the vast majority indicated support for student mental health, physical health, and academic issues while the few student parent support websites focused mostly on practical support, such as finding childcare and financial aid (Holmes and Nikiforidou, 2023).

### Individual

The coping resources section of the survey focuses on the ways that these student parents dealt with the changes they faced with the pandemic. It is important to know what they did or did not do or have access to in order to provide suggestions on what should be implemented at a university level to help student parents remain successful with schoolwork and research if another major event like the pandemic happens in the future. Within the SEM, the individual level will be greatly impacted when it comes to coping resources, their availability, and quality. Coping can be defined as something that individuals can use either as an activity, mindset, or action which allows them to manage the stressors of different situations (Skibniewski-Woods, 2022). If an individual does not find a positive way to cope, then they can experience burnout and decline mental health (Wilson et al., 2021). These parents discussed how they did not have the time or energy to stick to their normal routines, so it can be inferred that this may have included self-care as well. Positive coping strategies have been reported by student parents during the pandemic, such as prioritizing tasks, talking with others, self-care, positive thinking, and their religion (Holmes and Nikiforidou, 2023). Resources and suggestions such as these should be not only available to student parents, but they need to be well-known and widely advertised so that they can be used effectively.

### Relationship

Coping resources on the relationship level of the SEM refer to social support and resources that are available to individuals within their relationships. Relationships can include family, friends, professors, advisors, and their child/ren’s school teachers. One of the most important types of resources for this category is emotional support such as validation of feelings, encouragement, active listening, and reassurance. COVID-19 caused strain in many different relationships, and the student parents in the survey answered questions relating to the previously mentioned relationships. When asked the question, “While working from home, my research advisor was understanding of delays or unmet deadlines due to childcare obligations or disruptions,” over half of the responses were “strongly disagree.” Few of the respondents felt like their research advisor communicated effectively on a regular basis. Perhaps this is due to similar strains the research advisor was experiencing due to balancing their academic career and parenthood during the pandemic (Fulweiler et al., 2021). Positive communication skills include one’s ability to express themselves, actively listen, and provide feedback to the individual they are talking to, and this is something that many of these student parents were not receiving from their advisors during the pandemic. The survey included questions challenging whether or not these situations changed when they arrived back in person. Although there were slightly fewer “strongly disagree” responses than there were from the working from home questions, less-than-ideal support was provided that these student parents needed. The relationship between the student parent and their partner/spouse/significant other is essential to understand as well. If these student parents felt as though they were receiving more help during the transitions that COVID-19 threw at them, then they might have been able to feel more confident in battling the changes. However, 55% of the student parents strongly disagreed with the statement that their partner was able and/or willing to help with childcare while working from home. Seventy four percent of the respondents indicated that they either strongly or somewhat disagree with the statement that they felt their partner contributed to balancing childcare obligations during the pandemic. Sallee and Yates stress the importance of women-comprised networks to help specifically student mothers cope, bond, and persist throughout the pandemic because these internal support systems could step up when higher education institutions failed to do so (Sallee and Yates, 2023).

### Community

It is understood that being a student parent can be a challenging task, but there should be sufficient coping resources available on the community level to help these student parents succeed in their aspirations. One of the most common of those resources is community and institutional parent supporting groups. The open-ended question of “What type of resources does your institution provide for student parents?” received statements such as:

“none”

“very, very little”

“a smile and a wave”,

and

“outside of normal student resources, there are not any specialized for parents.”

These statements are also supported by the student support university website analysis performed by Holmes and Nikiforidou (2023). This is important because the lack of resources at most institutions causes individuals to feel unsupported and forgotten, or as Todd coined “invisible” (Todd, 2023). “Strongly disagree” was also the most popular choice on the survey question of whether their educational institution has provided adequate support/resources to student parents throughout the duration of the COVID-19 pandemic. This is unfortunate considering the fact that these student parents were forced to change the way that they learned in order to protect themselves, their families, and individuals at the school from COVID-19.

### Societal

Societal influences can play a major role in helping (or impeding) student parents in how they deal with the demands of childcare and schoolwork. There are a variety of coping resources that have been argued to be necessary for helping student parents, one of which is affordable childcare (Cruse et al., 2021; Williams et al., 2022). Access to reasonably priced and high-quality childcare, especially available on-campus, can help these parents have a sense of security financially and mentally (Cruse et al., 2021; Richardson, 2022; Williams et al., 2022). Another important aspect of this level is flexibility in academic schedules (Todd, 2023). Many student parents stated that they did not feel confident in completing their thesis and dissertation on time due to the pandemic. Having flexible academic and research schedules during the time that they were sent online and transitioning back in person would allow these individuals to balance their parental responsibilities and academic ones as well (King, 2022). Todd’s findings corroborate our results, demonstrating that student parents need flexibility in both time and space, as well as a sense of belonging through understanding, support, reassurance, and connection in order to sustain student parent success while at university (Todd, 2023). Further, when the pandemic hit, many individuals rushed into stores and bought high volumes of necessities for storage which caused an increase in price due to high demand. Such food price increases, subsequent supply chain issues, and fear of grocery shopping contributed to student food insecurity during the pandemic (Owens et al., 2020). The student parents in our survey did not have any positive comments to include when it comes to receiving aid or support at the societal level.

### Recommendations

The open-ended section of our survey allowed student parents to provide their own recommendations for how higher education institutions can help them navigate these challenges. The majority of student parents implore higher education institutions to establish procedures for flexible deadlines. One student parent asks that they

“allow for more flexibility in support and accommodations to help people in their unique situations.”

Another student parent writes,

“Student parents need so much grace and understanding. Clear and universal policies that are student/parent centered.”

The implementation of clear policies and procedures for flexible deadlines would help ensure that student parents can perform to the best of their ability in the case that childcare-related challenges arise. Further, student parents also suggest better clarity of the policies in place for student parents and that higher education institutions attempt to better understand the situations they face. One student parent requests,

“Better clarity and knowledge when it comes to what ways the university can be flexible with us and make those policies universal vs instructor by instructor, better understanding of what parents are actually dealing with these childcare interruptions continuing to happen, universal ability to allows students parents work from home as needed vs leaving it up to each individual supervisor. My supervisor is incredibly understanding but not all are.”

In addition to flexible procedures and clear policies, the majority of student parents also recommended that institutions provide on campus childcare centers. One student parent writes,

“Offer affordable on campus childcare!”

Similarly, another student parent writes,

“Offer childcare on campus. Have spaces for children on campus. Make resources more available and advertised.”

Lastly, a student parent writes,

“If not already available, I would think on-campus childcare would be helpful. As would a children-welcome parent support group (in person).”

On-campus childcare would eliminate the need for outside childcare and aid in logistics for attending classes by parents. However, while on-campus childcare has been offered at some institutions, faculty are usually prioritized for already competitive spots, and tuition is charged at the market rate (which is sometimes not affordable for faculty, let alone students with limited to no income) (Cruse et al., 2021). On-campus childcare should be carefully considered for implementation by higher education institutions to protect and maintain accessibility for financially insecure students, perhaps with a sliding-scale tuition based on income and/or in combination with professional teacher development at the institution (Barbour and Bersani, 1991; Richardson, 2022). Further, student parents also recommended that higher education institutions codify protections for student parents who need to miss class for childcare-related reasons. This protection would ensure that student parents are not at a disadvantage compared to their non-student parent counterparts due to circumstances outside of their control, such as a child falling ill.

Overall, it needs to be made abundantly clear to higher education institutions that a significant portion of their student population also has parenting responsibilities; it is not acceptable for over 20% of the student population to be “invisible” (Todd, 2023). Higher education institutions should also be made aware of the unique issues student parents face and enact policies and procedures to help alleviate the strain placed on them to make higher education more inclusive and accessible to this population. In conjunction with conveying this knowledge to these administrations, tangible support and resources need to be implemented. Holmes and Nikiforidou recommend enhanced availability of support for student parents, including designated support programs and counselors for student parents, improving the ethos of student parents among university constituents, and a review of policies to permit greater flexibility for student parents (Holmes and Nikiforidou, 2023). We also recommend enhanced visibility and advertisement of resources for student parents and for those resources to be more than superficial links to websites. There should be tailored support and resources for student parents at all stages of parenthood, including pregnancy (Holmes and Nikiforidou, 2023). Such resources should include conveying what rights student parents have to receiving an education; understanding Title IX guidelines for parenting students; how to apply for academic accommodations; how to find childcare in the area; finding and applying for insurance for giving birth and for children after birth, perhaps in the form of Medicaid or other state-funded children insurance programs; housing resources; food banks; how to apply for the women, infants, and children (WIC) nutrition program and other aid; study skill counseling; mental services counseling; time management counseling; tutoring services; and perhaps the most widespread, fostering and sustaining a visible student parent community (Fershee, 2010; Mason and Younger, 2014; U.S. Department of Education, 2020; Holmes and Nikiforidou, 2023). In addition, there are donor-supported programs (not funded by the university) such as “Baby Steps” at Auburn University, University of Alabama, University of Central Florida, and University of Tennessee that not only support students with unplanned pregnancies, but provide housing, childcare, meals, counseling, tutoring, and a number of other services for student mothers attending those institutions at no cost (Baby Steps, 2023).

However, how effective are such policies at higher education institutions? In 2016, Moreau identified three approaches higher education institutions use to support student parents, which include the careblind/universal, target, and mainstreaming approaches (Moreau, 2016b). The careblind/universal approach categorizes an institution as not having any specific provisions or policies in place, with an emphasis on universal policies needing to be equal for every student (Moreau, 2016b). The targeted approach has specific policies in place for student parents, such as specialized financial aid options or campus childcare (Moreau, 2016b). The mainstreaming approach attempts to make the needs of student parents more well-known and integrated into student policies, such as having designated websites or advisors for student parent support (Moreau, 2016b). Unfortunately, even with targeted or mainstream strategies, student parents report that they are largely unaware of the policies and/or do not have access to the resources the institution is attempting to provide (Moreau, 2016b; Baddley, 2021). In the report “Perception is Reality,” the Feminists for Life of America purport that while many higher education institutions have some level of on-campus resources for pregnant and parenting students, the basic resources are usually unpublicized (Utley, 2013; Baddley, 2021). Thus, higher education administrations need be more intentional in not only establishing such supportive resources for student parents, but being effective in their advertisement and follow-through of support.

## Conclusion

In conclusion, this study only begins to understand the unique challenges and situations student parents faced during the COVID-19 pandemic and their gradual return to in-person operations. While many of the isolating practices (remote work, remote school, hybrid teaching) that produced significant levels of stress are being rolled back, the aftermath of that stress and its long-term consequences remain to be fully understood. Addressing some of the challenges identified in this data will require further investigation at larger scales. This study provides an initial, cursory, exploratory glance at emerging complications of the COVID-19 pandemic for a particularly vulnerable group of students within higher education.

## Data Availability

The raw data supporting the conclusions of this article will be made available by the authors, without undue reservation.
